# Supplementary material for: Pyruvate Dehydrogenase Kinase Inhibition by Dichloroacetate in Melanoma Cells Unveils Metabolic Vulnerabilities
Source: Int J Mol Sci. 2022 Mar 29;23(7):3745. doi: 10.3390/ijms23073745 (PMC8999016; doi:10.3390/ijms23073745)
Supplement: Supplementary file 1 [file ijms-23-03745-s001.zip › ijms-1599788-supplementary.pdf]

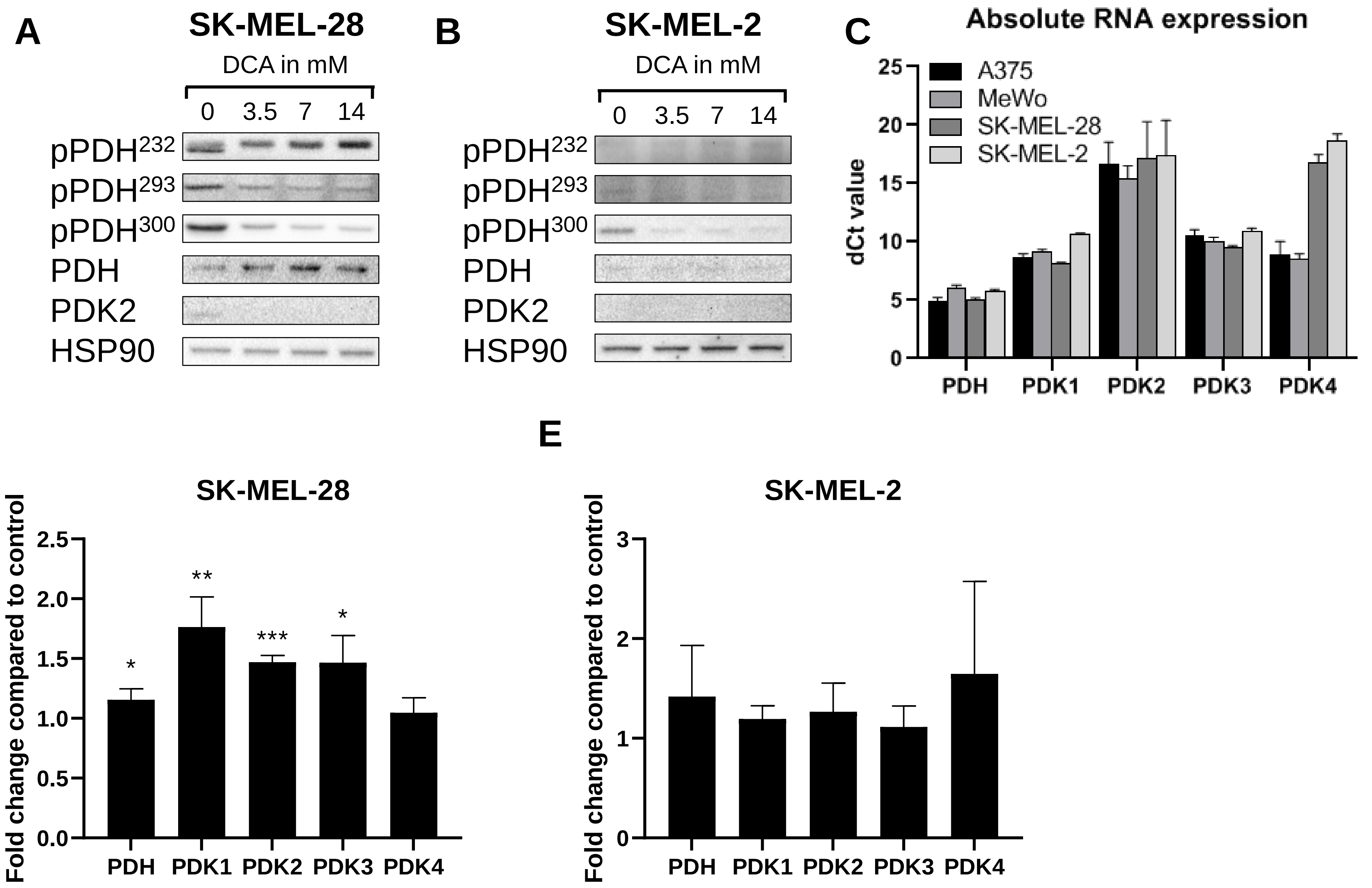

Figure S1. Effect of PDK inhibition on PDH and PDK expression in melanoma cells. Representative images of protein levels after 24h treatment with DCA in (**A**) SK-MEL-28 and (**B**) SK-MEL-2. (**C**) Absolute RNA levels (Ct value of target gene minus Ct value of β-actin loading control) in all cell lines. Fold change in RNA levels of PDH and PDK1-4 after 24h treatment with 14 mM DCA in (**D**) SK-MEL-28 and (**E**) SK-MEL-2. (**C-E**) Data represent mean ± SD of 3 independent experiments, each performed in quadruplicate. Student's *t*-test, * $p < 0.05$, ** $p < 0.01$, *** $p < 0.001$, DCA treated vs. control.

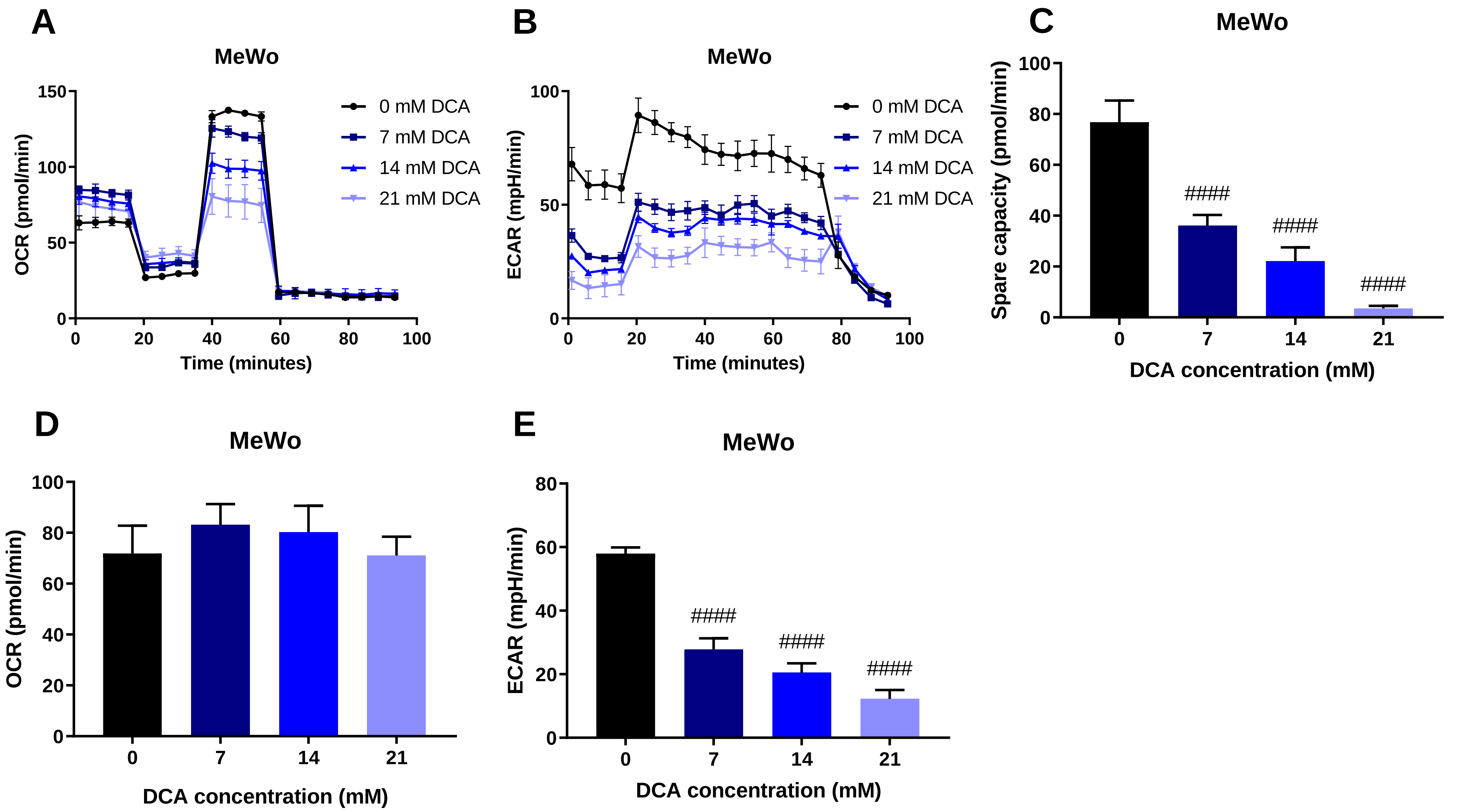

Figure S2. Metabolic adaptation upon PDK inhibition in MeWo cells. (**A**) Representative image of OCR measured by Seahorse, mean ± SD of 6-8 technical replicates are shown. (**B**) Representative image of ECAR measured by Seahorse, mean ± SD of 6-8 technical replicates are shown. (**C**) Spare capacity, e.g., maximal OCR minus basal OCR is decreased by DCA treatment. DCA treatment does not affect (**D**) basal OCR whereas it dose-dependently decreases (**E**) ECAR. (**C-E**) Data represent mean ± SD of 3 independent experiments, each consisting of 6-8 technical replicates. One-way ANOVA followed by Dunnett's multiple comparison test, #### p <0.0001, DCA treated vs. control.

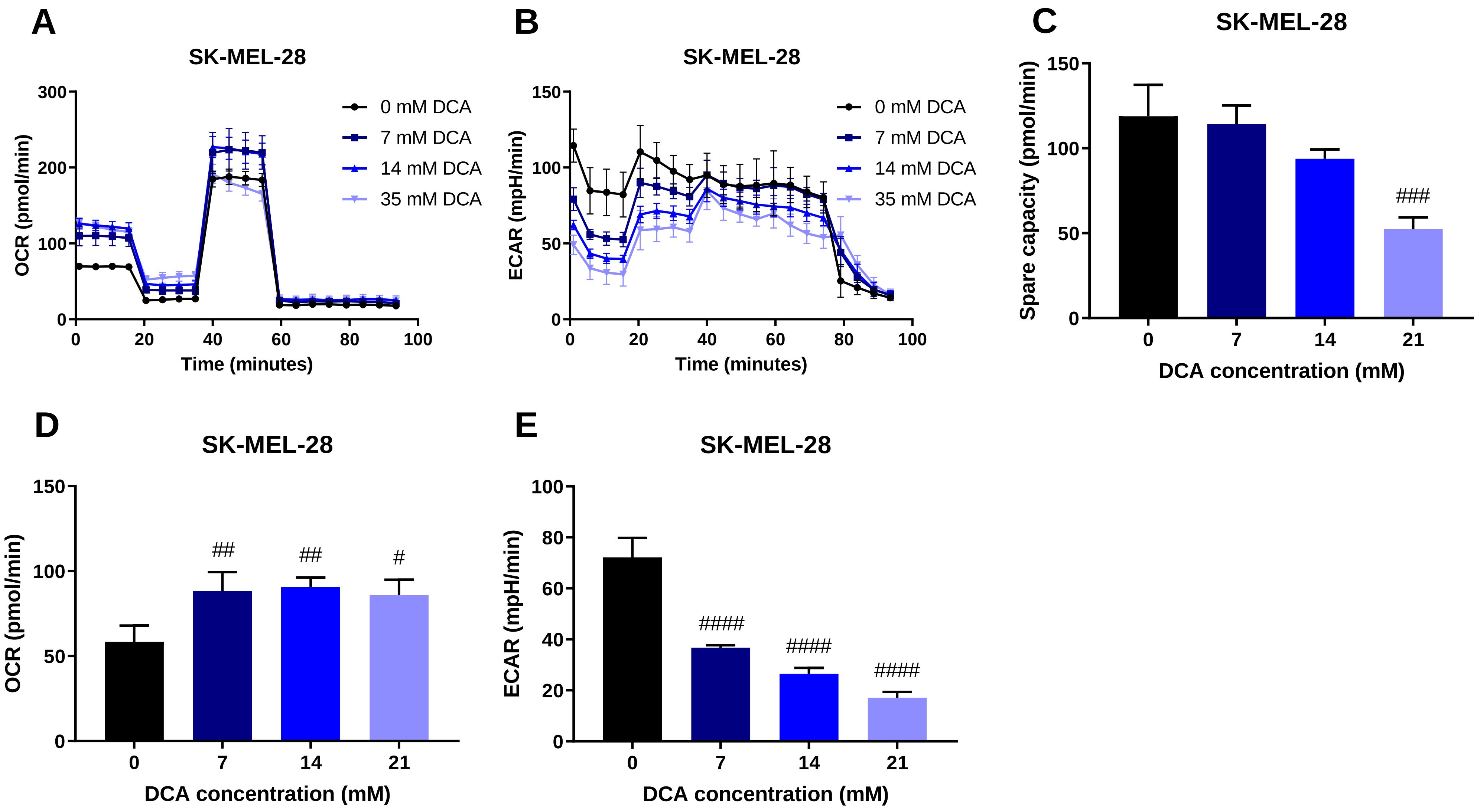

Figure S3. Metabolic adaptation upon PDK inhibition in SK-MEL-28 cells. (**A**) Representative image of OCR measured by Seahorse, mean ± SD of 6-8 technical replicates are shown. (**B**) Representative image of ECAR measured by Seahorse, mean ± SD of 6-8 technical replicates are shown. (**C**) Spare capacity, e.g., maximal OCR minus basal OCR is decreased by DCA treatment. DCA treatment increases (**D**) basal OCR and dose-dependently decreases (**E**) ECAR. (**C-E**) Data represent mean ± SD of 3 independent experiments, each consisting of 6-8 technical replicates. One-way ANOVA followed by Dunnett's multiple comparison test, # $p < 0.05$, ## $p < 0.01$, ### $p < 0.001$, #### $p < 0.0001$, DCA treated vs. control.

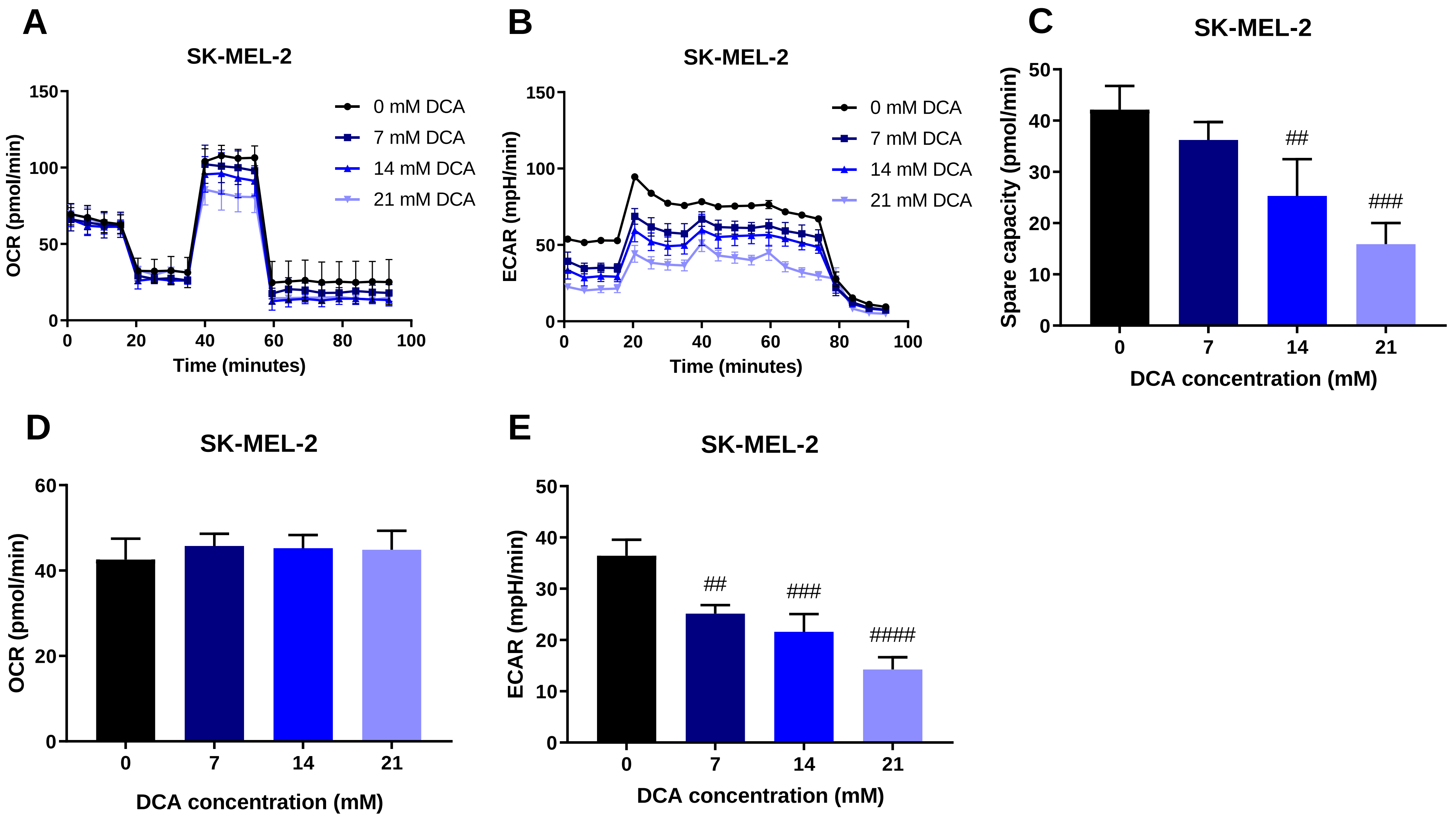

Figure S4. Metabolic adaptation upon PDK inhibition in SK-MEL-2 cells. (**A**) Representative image of OCR measured by Seahorse, mean ± SD of 6-8 technical replicates are shown. (**B**) Representative image of ECAR measured by Seahorse, mean ± SD of 6-8 technical replicates are shown. (**C**) Spare capacity, e.g., maximal OCR minus basal OCR is decreased by DCA treatment. DCA treatment does not affect (**D**) basal OCR whereas it dose-dependently decreases (**E**) ECAR. (**C-E**) Data represent mean ± SD of 3 independent experiments, each consisting of 6-8 technical replicates. One-way ANOVA followed by Dunnett's multiple comparison test, ## $p < 0.01$, ### $p < 0.001$, #### $p < 0.0001$, DCA treated vs. control.

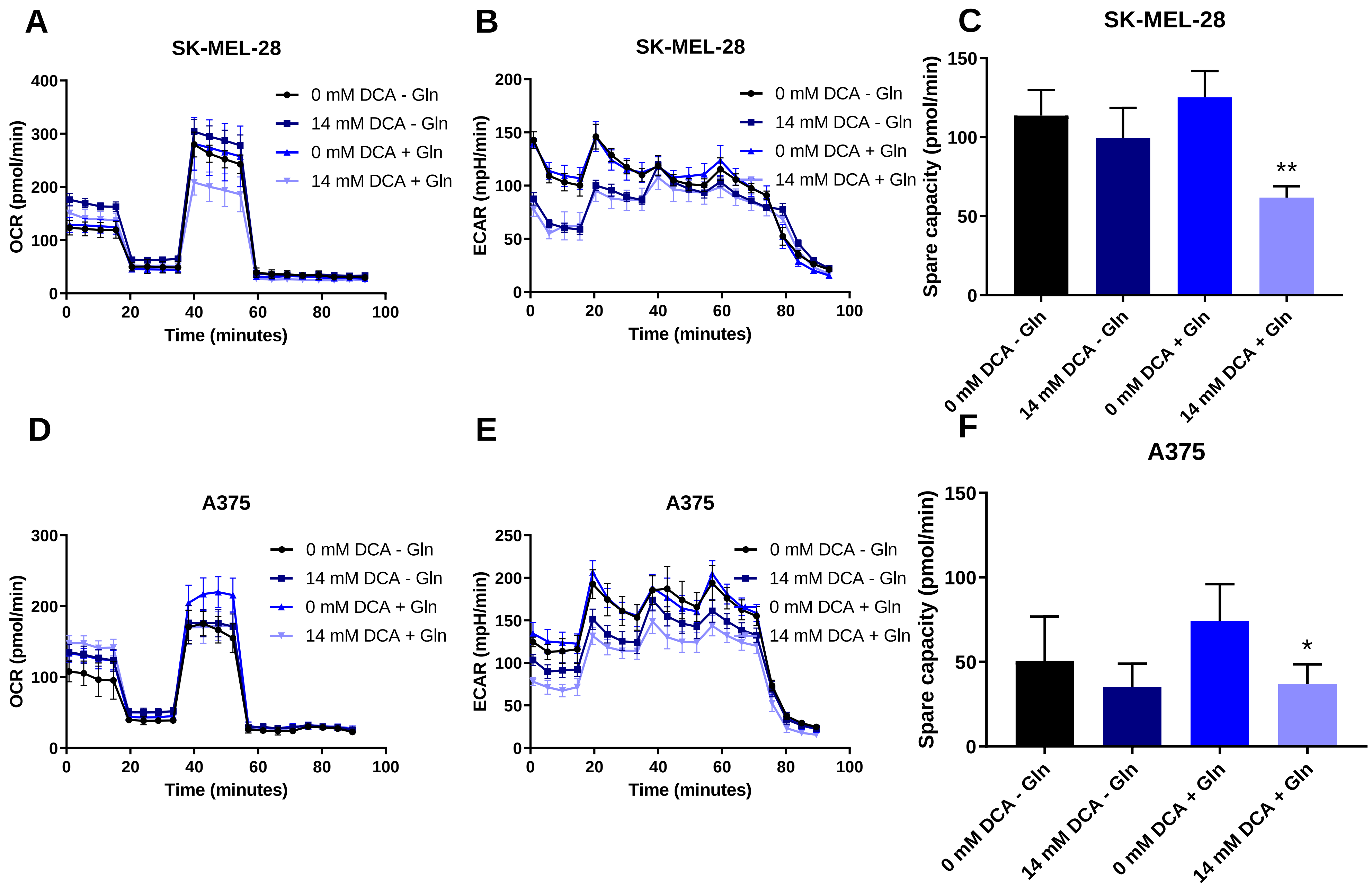

Figure S5. Effect of glutamine in medium on DCA response. Representative image of OCR measured by Seahorse in (**A**) SK-MEL-28 and in (**D**) A375, mean ± SD of 6-8 technical replicates are shown. Representative image of ECAR measured by Seahorse in (**B**) SK-MEL-28 and in (**E**) A375 , mean ± SD of 6-8 technical replicates are shown. Spare capacity is decreased upon DCA treatment in glutamine-containing, but not in glutamine-free medium in both (**C**) SK-MEL-28 and in (**F**) A375. (**C**,**F**) Data represent mean ± SD of 3-4 independent experiments, each consisting of 6-8 technical replicates. Student's *t*-test, * $p < 0.05$, ** $p < 0.01$, DCA treated compared to control.

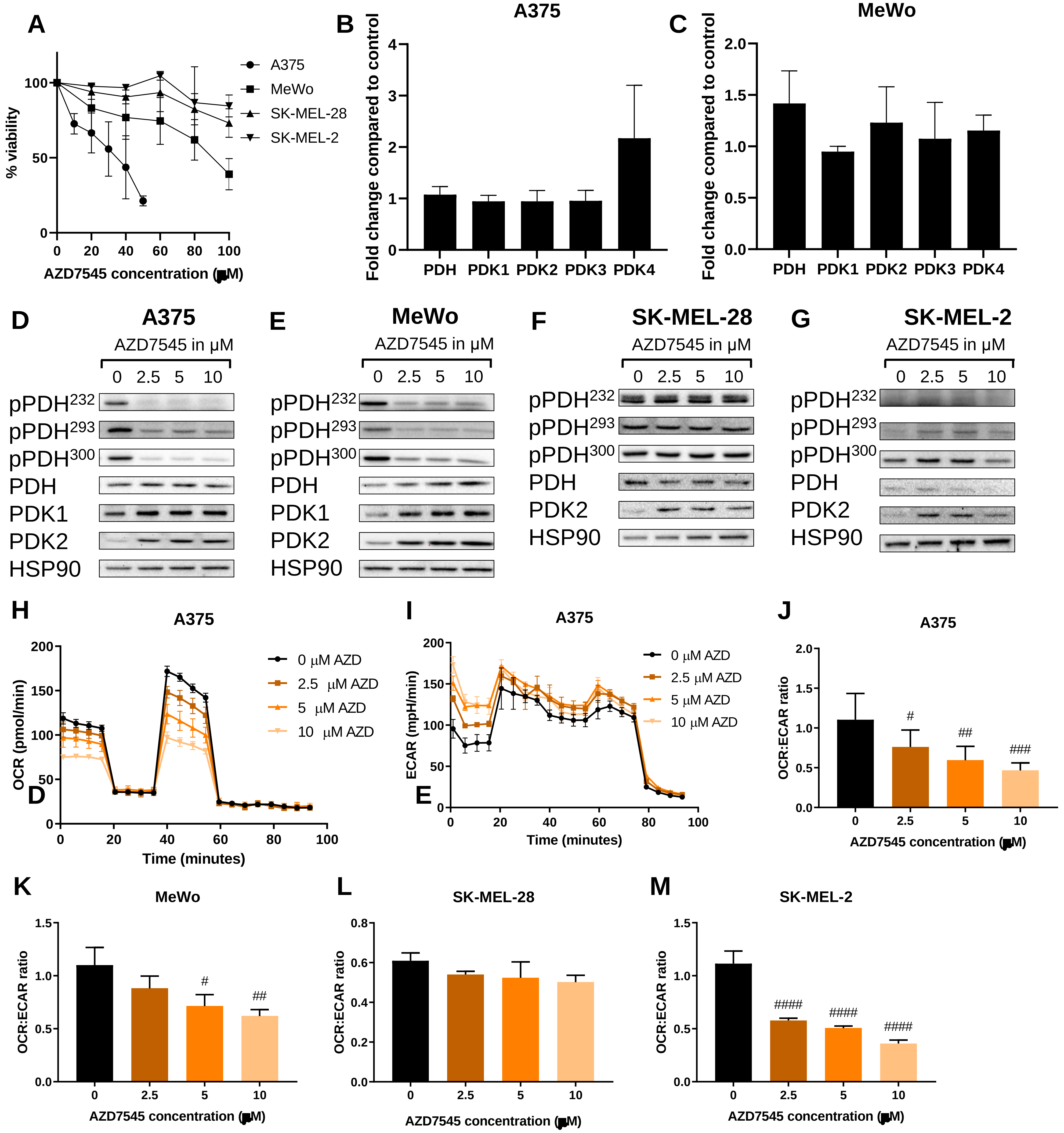

Figure S6. Effect of AZD7545 on melanoma cell lines. (**A**) Viability after 24h of AZD7545 treatment. Fold change in RNA levels of PDH and PDK1-4 after 24h treatment with 10 µM AZD7545 in (**B**) A375 and (**C**) MeWo. Representative image of protein levels after 24h treatment with AZD7545 in (**D**) A375, (**E**) MeWo, (**F**) SK-MEL-28 and (**G**) SK-MEL-2. For the $pPDH^{232}$ protein, a double band is visible in the SK-MEL-28 cell line: the lower band is the band of interest. (**H**) Representative image of OCR measured by Seahorse, mean ± SD of 6-8 technical replicates are shown. (**I**) Representative image of ECAR measured by Seahorse, mean ± SD of 6-8 technical replicates are shown. AZD7545 treatment decreases OCR:ECAR ratio in (**J**) A375, (**K**) MeWo and (**M**) SK-MEL-2 but not in (**L**) SK-MEL-28 cells. (**A-C**) Data represent mean ± SD of 3 independent experiments, each consisting of 3-4 technical replicates. (**J-M**) One-way ANOVA followed by Dunnett's multiple comparison test, # $p < 0.05$, ## $p < 0.01$, ### $p < 0.001$, #### $p < 0.0001$, AZD7545 treated vs. control.

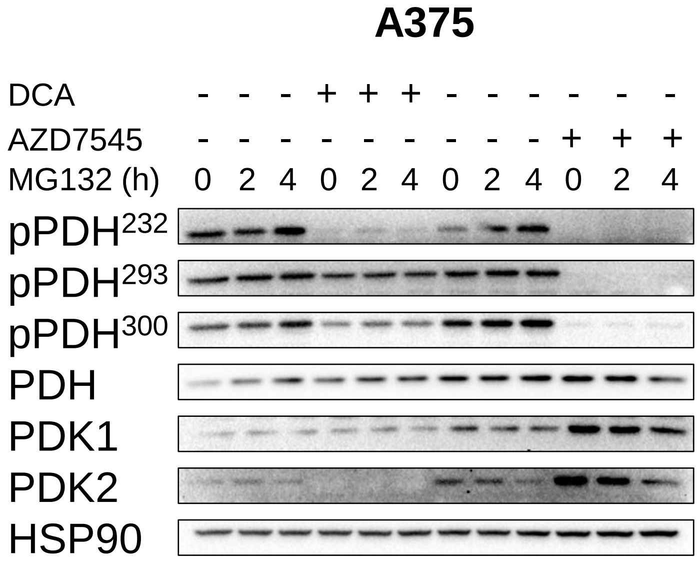

Figure S7. Neither DCA nor AZD7545 treatment leads to a difference in protein production. Representative image of protein levels in A375 cells after treatment with 10 μM proteasome inhibitor MG132 for up to 4 hours with and without DCA or AZD7545. DCA is dissolved in H_20, whereas AZD7545 is dissolved in DMSO, hence the use of two controls.

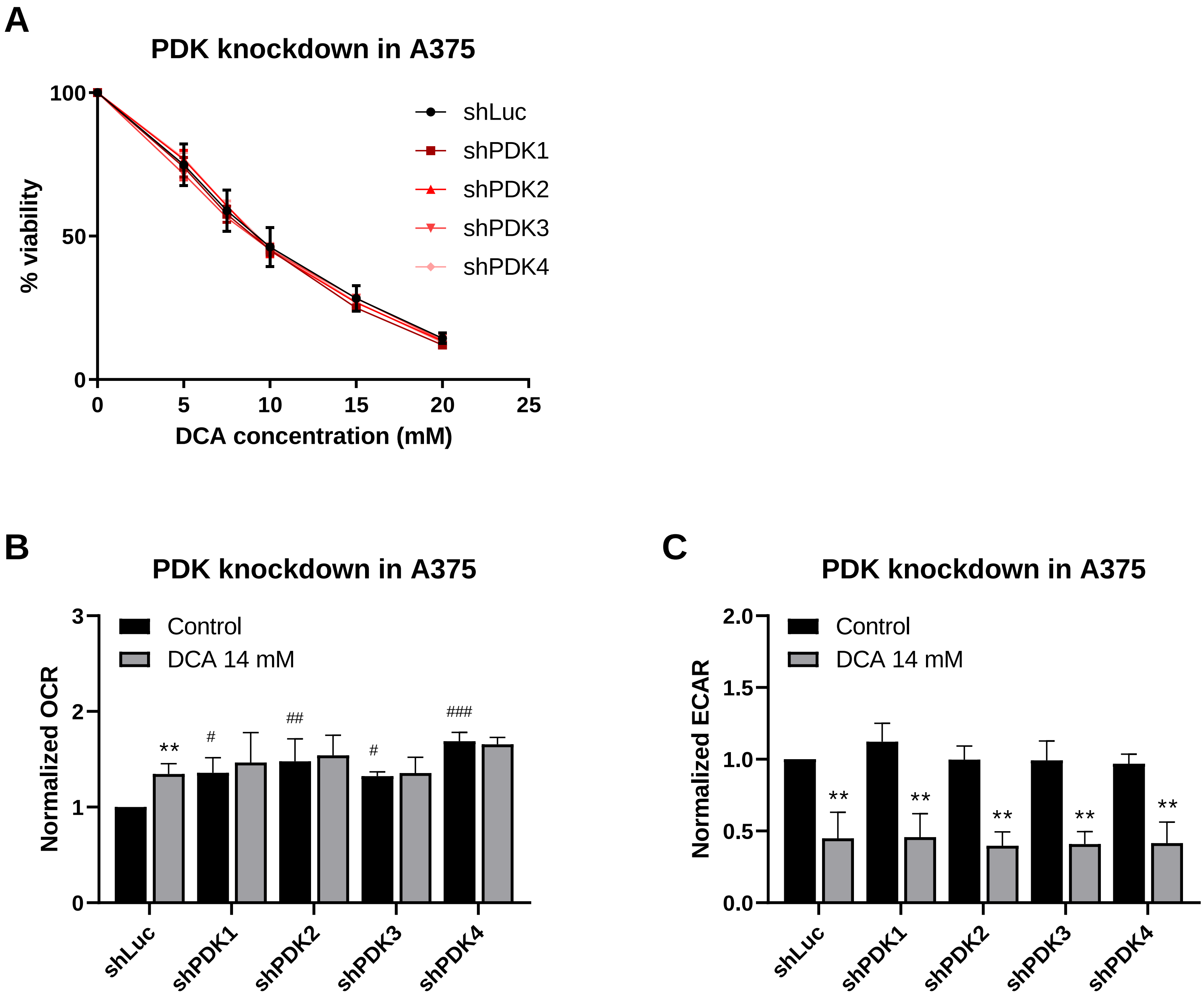

Figure S8. Effect of PDK knockdown on viability, metabolic phenotype and response to DCA. (**A**) Sensitivity of A375 cells to DCA does not change after PDK knockdown. OD values shown in Figure 4F were normalized to the untreated cells of each knockdown. Data represent mean ± SD of 3 independent experiments, each performed in triplicate. (**B**) PDK knockdown led to an increase in OCR with little to no additional effect of DCA. (**C**) PDK knockdown did not lead to differences in ECAR, in contrast to DCA treatment. Data represent mean ± SD of 3 independent experiments, each containing 3-8 technical replicates. (**A**) No significant differences were observed. (**B**,**C**) One-way ANOVA followed by Dunnett's multiple comparison test, # $p < 0.05$, ## $p < 0.01$, ### $p < 0.001$, shPDK1-4 vs. shLuc (black bars). Student's *t*-test, ** $p < 0.01$, DCA treated vs. control (grey bars).

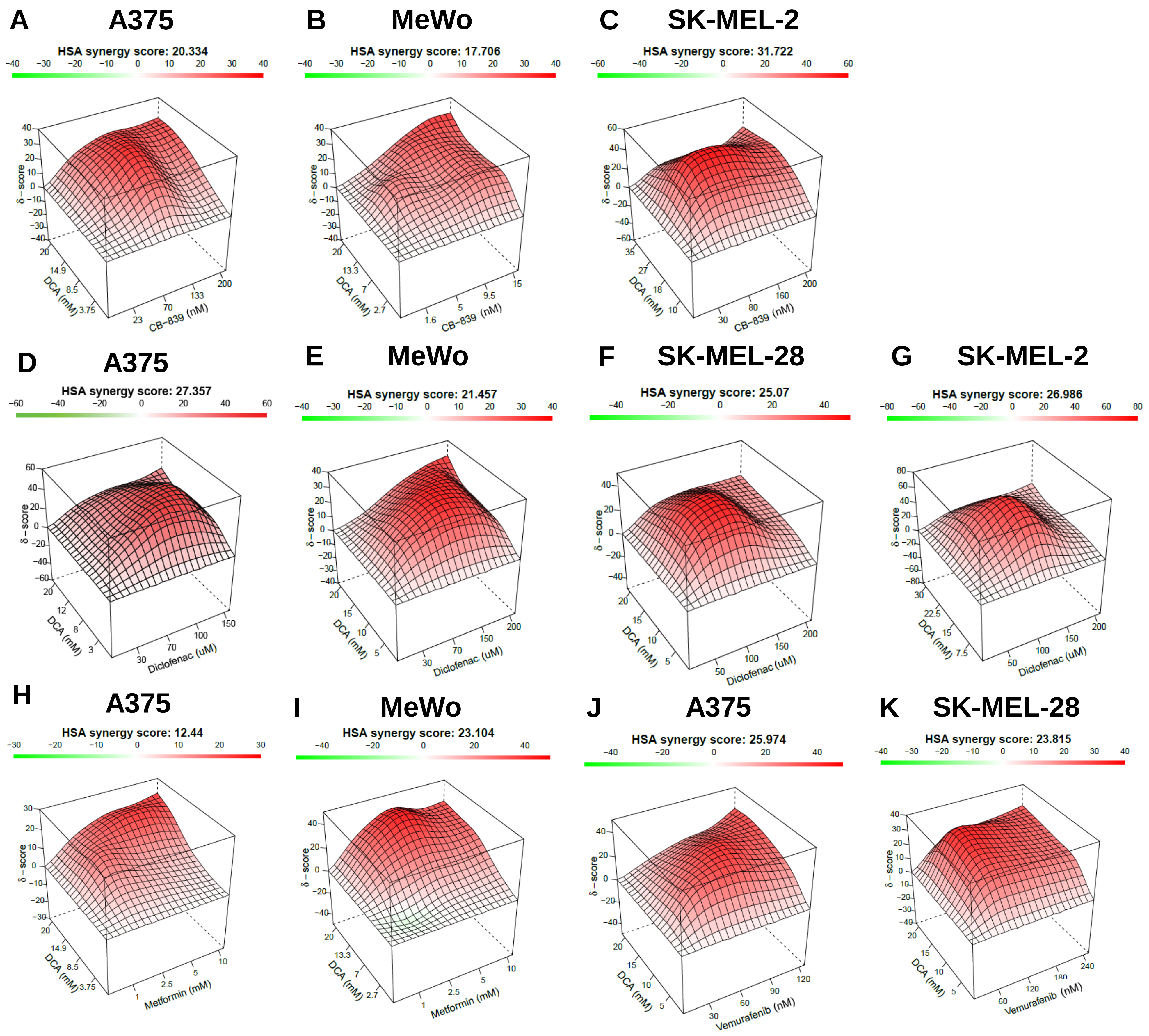

Figure S9. Synergy scores of DCA combined with other metabolic inhibitors. DCA in combination with CB-839 showed synergy in (**A**) A375, (**B**) MeWo and (**C**) SK-MEL-2. (**D**-**G**) DCA in combination with diclofenac showed synergy in all four cell lines. DCA in combination with metformin showed synergy in (**H**) A375 and (**I**) MeWo. DCA in combination with vemurafenib showed synergy in (**J**) A375 and (**K**) SK-MEL-28. Synergy is assumed with values above 10, where the value represents the average excess response of two drugs, i.e., with a synergy score of 20, 20% of the response can be attributed to drug interactions and not addition alone. Each experiment was carried out in triplicate and the mean ± SD of 3-6 experiments are shown.

Table S1. Forward and reverse sequences for primers used in this study. All are manufactured by Eurogentec.

Gene	Forward sequence	Reverse sequence
PDH	GGATGGTGAACAGCAATCTTGCC	TCGCTGGAGTAGATGTGGTAGC
PDK1	CAGGACAGCCAATACAAGTG	GTTGGCATGGTGTTCCATAG
PDK2	CTATCTCAAGGCCCTGTC	TCCTGGATGGTCTGGTAG
PDK3	CCAGAGCTGGAAGTTGAAG	GTGAGGGCACATAAACCAC
PDK4	AAGCCCAGATGACCAGAAAG	TGATTGGTGACTGGGTCAAC
ACTB	ACTCTTCCAGCCTTCCTTCC	CAATGCCAGGGTACATGGTG

Table S2. shRNA sequences for generation of PDK knockdown cell lines, courtesy of dr. Aysegül Erdem.

Gene	Sequence
Luciferase	CCGCCTGAAGTCTCTGATTAA
PDK1	TTAGAGACTGTGTTGTTAGTTA
PDK2	ATCCAGCAATGCCTGTGAGAAA
PDK3	ACAGGTCTTGGATAACTTTCTA
PDK4	ACACTATGTGGTTACAAATATA
